# Tailoring of the desired selectivity and the turn-on detection range in a self-assembly-based fluorescence sensory system†
†Electronic supplementary information (ESI) available: Details of synthesis and spectroscopic data. See DOI: 10.1039/c5sc00863h



**DOI:** 10.1039/c5sc00863h

**Published:** 2015-05-06

**Authors:** Takao Noguchi, Bappaditya Roy, Daisuke Yoshihara, Youichi Tsuchiya, Tatsuhiro Yamamoto, Seiji Shinkai

**Affiliations:** a Institute for Advanced Study , Kyushu University , 744 Moto-oka, Nishi-ku , Fukuoka 819-0395 , Japan . Email: shinkai_center@mail.cstm.kyushu-u.ac.jp ; Email: tnoguchi@mail.cstm.kyushu-u.ac.jp ; Fax: +81-92-805-3814 ; Tel: +81-92-802-6990; b Nanotechnology Laboratory , Institute of Systems Information Technologies and Nanotechnologies (ISIT) , 4-1 Kyudai-Shinmachi, Nishi-ku , Fukuoka 819-0388 , Japan; c Department of Nanoscience , Faculty of Engineering , Sojo University , 4-22-1 Ikeda , Kumamoto 860-0082 , Japan

## Abstract

This study demonstrates how to control the selectivity and the turn-on detection range toward the tailoring of an assembly-based fluorescence (FL) sensory system. Assembly-based FL chemosensors composed of oligophenylenevinylene with a varied number of guanidinium receptors (G2, G4 and G6) were newly developed, and their FL response to nucleotides (AMP, ADP and ATP) was investigated. Indeed, G6 exhibited FL emission *via* self-assembly with ATP. More importantly, the FL response of G6 showed markedly improved selectivity for ATP over ADP and a broadly extended detection range of ATP concentration under adjusted salt conditions. The salt effect on the FL response revealed the competitive binding interactions affecting the subsequent self-assembly process. These studies have unveiled the pivotal binding mechanisms operating in the self-assembly process, which tailor the performance level of the assembly-based sensory system. We believe that this study offers a new design principle of an assembly-based FL chemosensor with high selectivity and the appropriate detection range, being different from the conventional key-and-lock system.

## Abstract 2

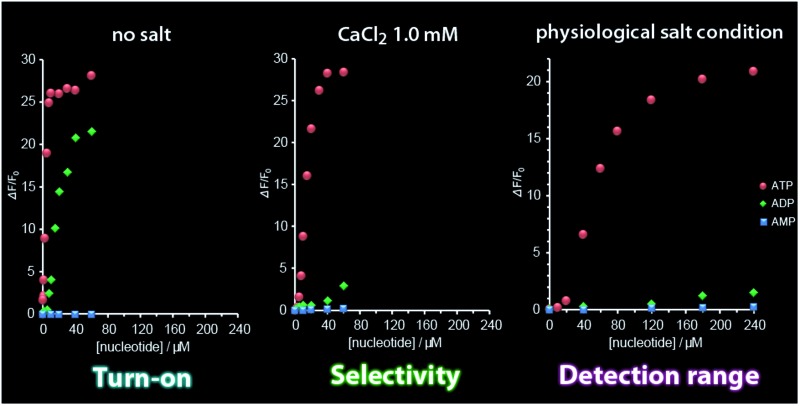
A new assembly-based fluorescent sensor exhibits much improved selectivity for ATP over ADP and a broad detection range under adjusted salt conditions, providing insight into a pivotal binding mechanism in the self-assembly process.

## Introduction

Ranging from natural to artificial supramolecular materials, noncovalent interactions determine the properties of their systems leading to their characteristic functions.^1,2^ For example, multiple hydrogen bonds in complementary nucleobase pairs construct and stabilize the double helix structure of DNA. Also, the relationship between a virus infection and the immune system is classified as a protein–ligand interaction realized through their noncovalent interactions. This idea of noncovalent binding mechanisms has been applied to the design and functionalization of supramolecular materials:^3–9^ for example, supramolecular polymers, functional gels and chemosensors. In order to attain functionalities, according to the key-and-lock concept,^10^ one may design a new artificial receptor for a target. Is it possible to develop a new strategy capable of tailoring the functionalities?

**Fig. 1:**
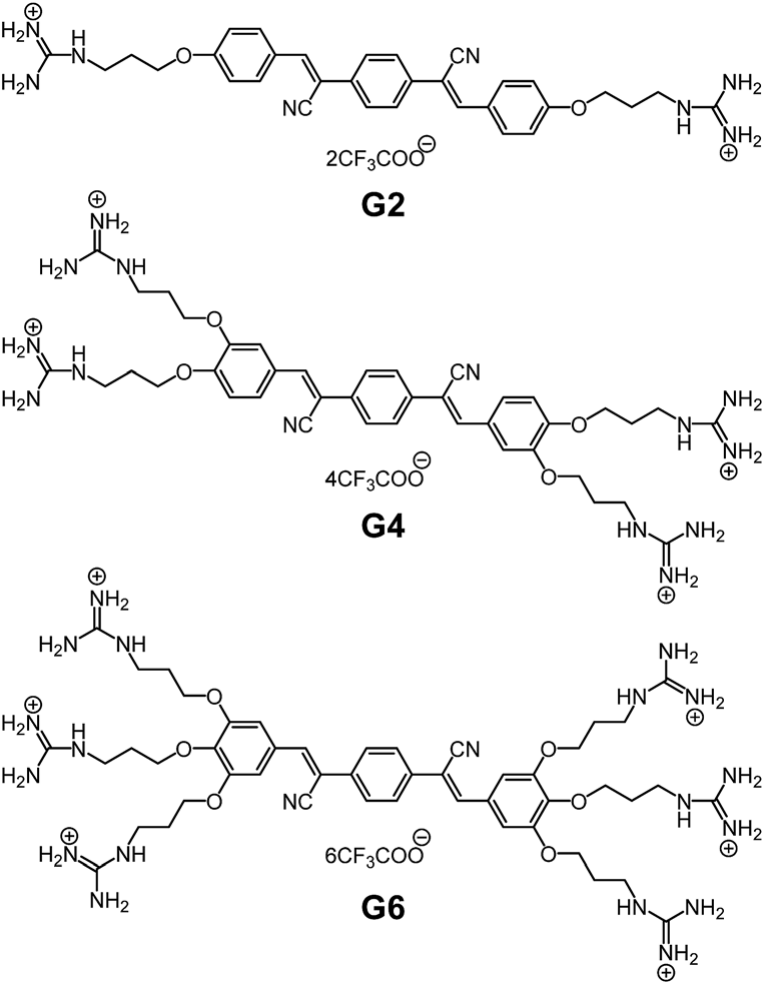
Chemical structures of G2, G4 and G6.

We have reported self-assembly-based fluorescence (FL) chemosensors by utilizing aggregation-induced emission (AIE).^11^ The advantage of AIE is that the FL emission is switched on upon the self-assembly of molecules through interaction with a targeted analyte.^12^ In this context, such an assembly-based FL system is inherently different from conventional systems based on molecular recognition through a key-and-lock-type receptor–guest binding.^13^ Recently, we have offered a new perspective that self-assembly works as a functional system to translate structural information on the target, *via* its self-assembly morphology, eventually into FL optical output.^14^ This translation cascade utilizing the self-assembly phenomena realizes the fluorometric detection of a subtle difference in the targeted molecular structures, thus unveiling the potential ability of self-assembly as a sensory system. In order to apply this concept as a practical sensory system, we must overcome the following one drawback specific to the self-assembly system: that is, FL emission arising from self-aggregation of the sensor molecule itself.^15^ In other words, the sensor molecule should be virtually nonfluorescent under various measurement conditions, while aggregation leading to FL emission should occur sensitively in the presence of the target molecule. We thus developed oligophenylenevinylene (OPV) sensors with a varied number of guanidinium receptors (G2, G4 and G6, shown in Fig. 1),^16,17^ and confirmed that G6 has a superior tolerance to its self-aggregation even under a high salt concentration. In the course of the study on its FL response to nucleotides (AMP, ADP and ATP), we found that the selectivity of G6 for ATP over ADP is much improved and that the detection range of ATP concentration is expanded under certain salt conditions. This is attributable to the result of the binding competition between the targets and the added salts. Herein we have gained a new insight into how to control the selectivity and the turn-on detection range in an assembly-based FL sensory system. This perspective is achieved when our attention is shifted from the conventional AIE phenomena to the binding events utilizing the self-assembly process. In this contribution, we wish to emphasize the importance of this binding mechanism in affecting the subsequent self-assembly process and the consequent performance level of the assembly-based FL sensory system.

## Results and discussion

### FL response of the OPV sensors to nucleotides

**Fig. 2:**
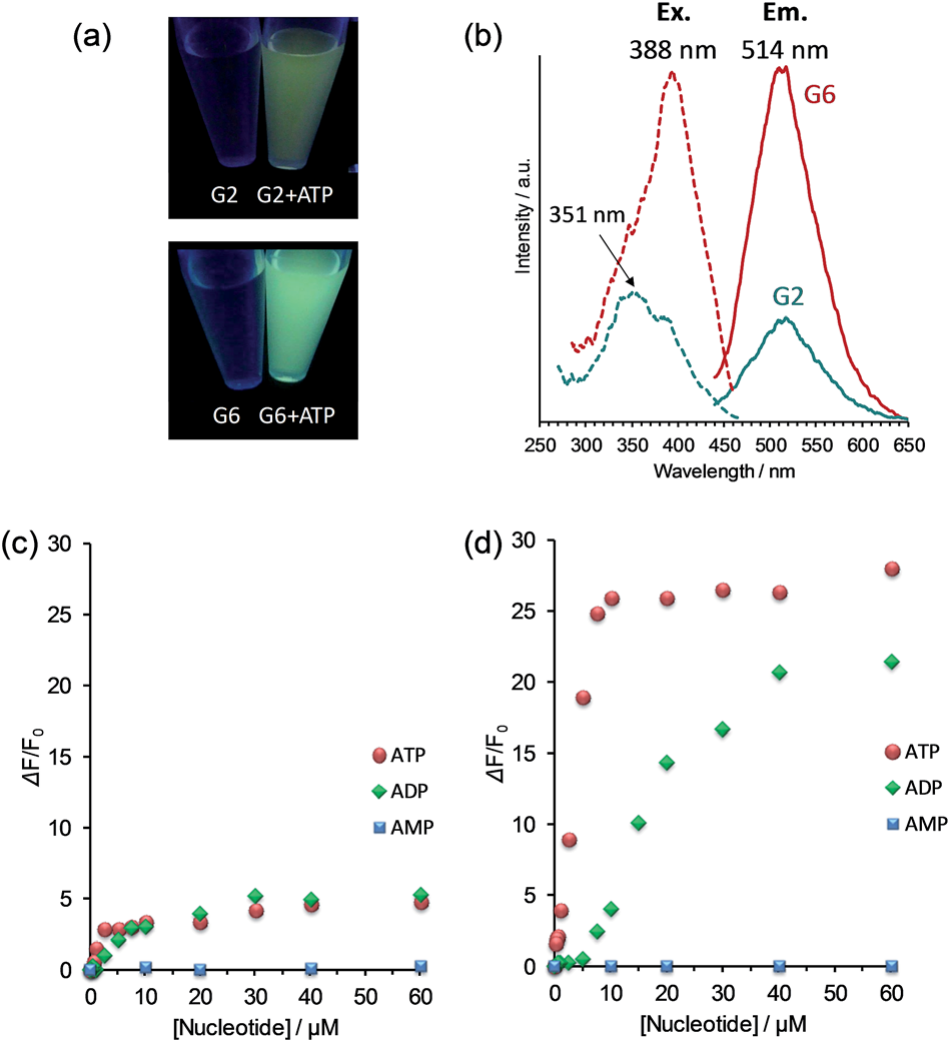
(a) Photographs of G2 and G6 (*λ*_ex_ = 365 nm) in the presence of ATP (20 μM). (b) Excitation (*λ*_em_ = 514 nm) and fluorescence spectra (*λ*_ex_ = 388 nm) of G2 and G6 in the presence of ATP (20 μM) in DMSO–water (1 : 9 v/v); green line: G2; red line: G6. Fluorescence titration curve of (c) G2 and (d) G6 upon addition of increasing concentrations of AMP (blue), ADP (green) and ATP (red) in DMSO–water (1 : 9 v/v). Conditions: [G2] = [G6] = 6.0 μM; [HEPES] = 10 mM (pH 7.4); 25 °C; *λ*_ex_ = 388 nm; *λ*_em_ = 514 nm.

A series of guanidinium-tethered OPV sensors (G2, G4 and G6) was synthesized according to the previously reported procedure (Scheme S1 in ESI†).^14^ G4 and G6 bearing four and six guanidinium groups, respectively, were water-soluble, whereas the addition of a small amount of DMSO was necessary to solubilize G2 in water (Fig. S1 in ESI†). Thus, the mixed solvent system of DMSO–water (1 : 9, v/v) was selected for the following measurements, and particular attention was paid to the difference between the two extreme cases of G2 and G6 in their FL responses to nucleotides induced by self-assembly.^18^ Although G2 and G6 are virtually nonfluorescent in this solvent, addition of ATP makes the FL turn-on with the FL intensity observable by our naked eyes (photographs in Fig. 2a, [G2] = [G6] = 6.0 μM, [ATP] = 20 μM). The FL emission maximum appeared at 514 nm, but a significant difference in the FL intensity was observed between G2 and G6 in spite of the same experimental conditions (Fig. 2b). In order to understand the origin of their FL behaviors, we performed a FL titration experiment. When the FL intensity was plotted against the ATP concentration, the FL intensity for G2 reached the maximum plateau where the FL increase was 5-fold (Fig. 2c and S3 in ESI†), while that for G6 reached the maximum plateau where the FL increase was 26-fold (Fig. 2d and S3 in ESI†). It is surprising that such a big difference in the FL intensity is observed when changing only the number of guanidinium groups. In fact, the FL intensity reflects the difference in the self-assembly tendency as proposed previously.^14^ This view is clearly supported by the present excitation spectra (Fig. 2b) where the relative intensity of the excitation bands at 351 and 388 nm is significantly different between G2 and G6.

### Self-assembly behavior of the OPV sensors governing the characteristic FL responses to ATP

We performed UV-Vis titration experiments in order to clarify the self-assembling behaviors and noticed that the UV-Vis spectra for G2 and G6 change quite oppositely upon addition of ATP. With the increase in the ATP concentration, the original absorption maximum of G2 at 374 nm decreased gradually and eventually shifted to 352 nm (Fig. S4a in ESI†). This spectral change is ascribed to the characteristic H-type stacking of OPV chromophores as reported in previous literature.^16*b*^ On the contrary, the absorption maximum of G6 at 371 nm shifted to the longer wavelength of 395 nm (Fig. S4b in ESI†). This shift to a longer wavelength can be rationalized in terms of a slipped stacking of the OPV chromophores with respect to the direction of the molecular long axis.^19^ Therefore, one can classify this mode of arrangement as J-type stacking. Such a difference in the mode of arrangement is reflected in their visual images in the self-assembly morphologies. The H-type aggregates of G2/ATP gave a finite morphology, whereas the J-type aggregates of G6/ATP gave a spherical particle-like morphology (Fig. S5 in ESI†). These results provide a clear view of the structure-based translational cascade as proposed previously.^14^ The association of G2 with ATP forms H-type stacked finite aggregates resulting in a modest FL intensity, whereas the association of G6 with ATP generates J-type stacked spherical aggregates leading to a strong FL intensity.

### Self-aggregation behavior of the OPV sensors under salt conditions

**Fig. 3:**
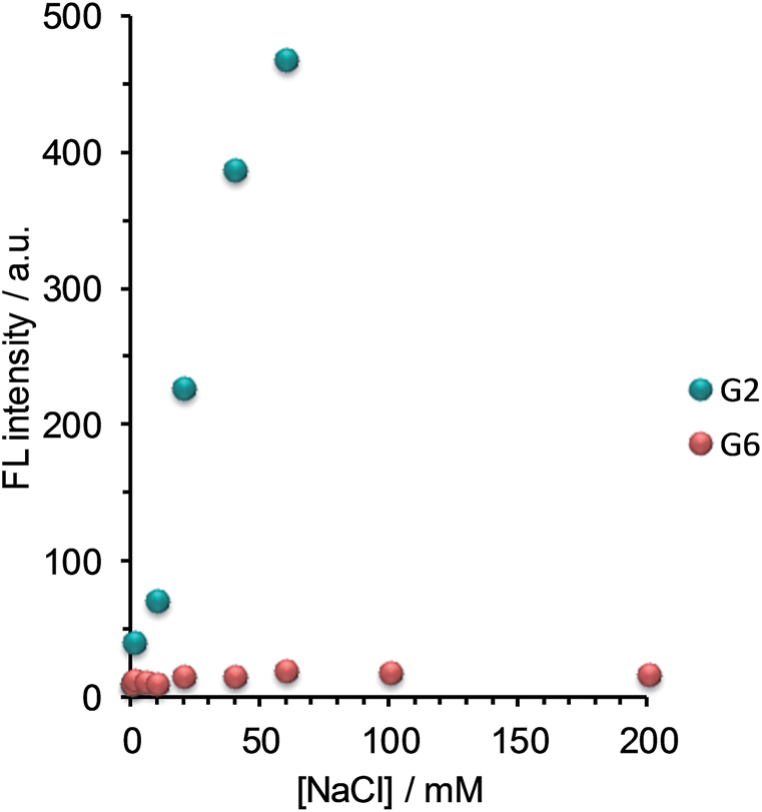
Fluorescence emission dependence on salt concentration arising from the self-aggregation of G2 (green) and G6 (red) in DMSO–water (1 : 9 v/v). Conditions: [G2] = [G6] = 6.0 μM; [HEPES] = 10 mM (pH 7.4); 25 °C; *λ*_ex_ = 388 nm; *λ*_em_ = 514 nm.

For the practical application of this assembly-based sensory system, it is desirable to suppress the FL emission arising from OPV self-aggregation under salt conditions. We first tested the influence of the salt concentration on the self-aggregation behavior of the OPV sensors ([G2] = [G6] = 6.0 μM), which is very important to determine as a step toward the practical application in biomedical assays. As shown in Fig. 3, the FL intensity of G2 increased with the increase in the NaCl concentration. This result clearly indicates that the self-aggregation of G2 is induced by the addition of only 10 mM NaCl. In contrast, no change in the FL intensity of G6 was observed even at 200 mM NaCl, indicating that the self-aggregation of G6 is not induced even under physiological salt conditions. Next, the influence of the OPV concentration on the FL intensity was investigated at the physiological salt concentration. As expected, the FL peaks of G6 were negligibly small up to 10 μM, indicating its superior tolerance to self-aggregation (Fig. S6 in ESI†). These results support a view that in the OPV sensors the increase in the charged group number is effective in increasing the tolerance to undesired self-aggregation even under physiological salt conditions.

### FL response of G6 to nucleotides under salt conditions

**Fig. 4:**
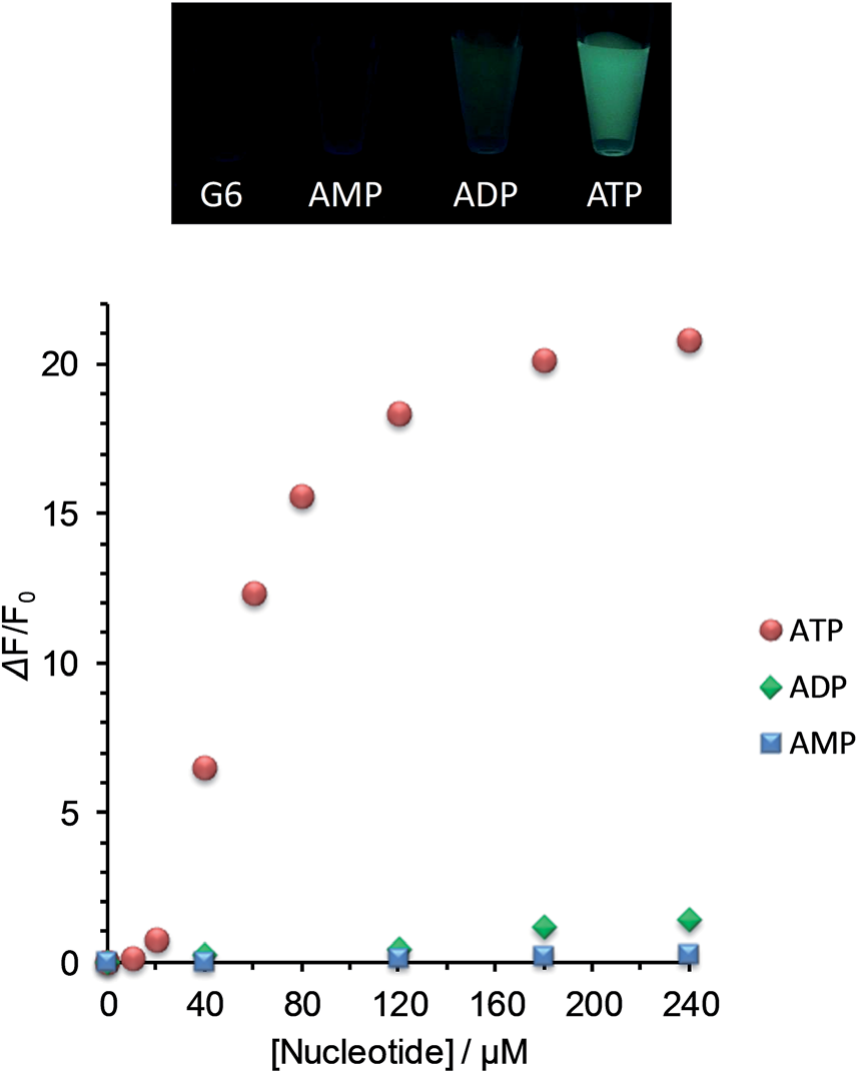
Photograph of G6 (*λ*_ex_ = 365 nm) in the presence of AMP, ADP and ATP (180 μM for the three) and fluorescence titration curves (*λ*_ex_ = 388 nm; *λ*_em_ = 514 nm) of G6 upon addition of increasing concentrations of AMP (blue), ADP (green) and ATP (red) under physiological salt conditions. Conditions: [G6] = 6.0 μM; [NaCl] = 125 mM; [KCl] = 5.0 mM; [CaCl_2_] = 1.0 mM; [MgCl_2_] = 0.5 mM; [HEPES] = 10 mM (pH 7.4); 25 °C.

As the first example for the practical application of G6, we explored its FL response to nucleotides under physiological salt conditions. We found that the selectivity of G6 for ATP over ADP is markedly improved under conditions of high salt concentration and that the turn-on detection range of ATP concentration is further expanded (Fig. 4 and S7 in ESI†).^20^ In addition, we confirmed that the FL response of G6 is efficiently decreased by the presence of ATPase (Fig. S9 in ESI†), undoubtedly supporting the view that the FL intensity is correlated with the ATP concentration. It is worth emphasizing here that this G6 sensor is applicable to enzyme activity assays owing to its preferential selectivity for ATP.

**Fig. 5:**
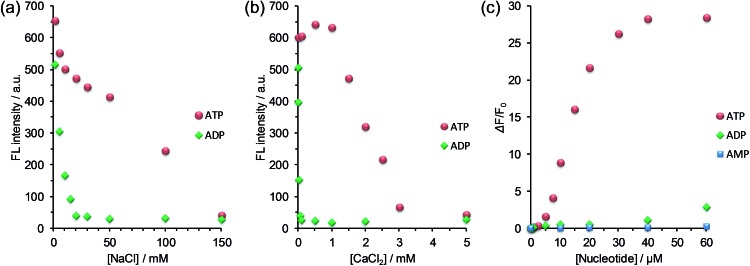
Changes in the fluorescence intensity of G6/nucleotide (green: ADP; red: ATP) upon addition of increasing concentrations of (a) NaCl and (b) CaCl_2_ in DMSO–water (1 : 9 v/v); conditions: [G6] = 6.0 μM, [nucleotide] = 20 μM, [HEPES] = 10 mM (pH 7.4), 25 °C; *λ*_ex_ = 388 nm; *λ*_em_ = 514 nm. (c) Fluorescence titration result of G6 upon addition of increasing concentrations of AMP (blue), ADP (green) and ATP (red) with a fixed concentration of CaCl_2_ (1.0 mM); conditions: [G6] = 6.0 μM; [HEPES] = 10 mM (pH 7.4); 25 °C; *λ*_ex_ = 388 nm; *λ*_em_ = 514 nm.

Here, a question newly born in our minds still remains: why is the selectivity improved and is the detection range expanded? In order to gain some insights, we estimated the salt effect on the FL response of G6 to ADP and ATP at fixed G6 and nucleotide concentrations ([G6] = 6.0 μM, [ADP] = [ATP] = 20 μM). With the increase in the NaCl concentration (0–150 mM), the FL intensity of G6/nucleotide was decreased owing to the competitive inhibition of the guanidinium–phosphate binding (Fig. 5a). The clear difference in the degree of FL intensity decrease between G6/ADP and G6/ATP is attributable to the difference in the anionic charge density between ADP and ATP. Namely, formally tetra-anionic ATP shows the stronger electrostatic interaction and the more tolerant property toward the competitive inhibition than tri-anionic ADP. With regard to the salt effects, a conspicuous effect observed for CaCl_2_ should be highlighted (Fig. 5b). With the increase in the CaCl_2_ concentration (0–1.0 mM), the FL intensity of G6/ATP remained at an almost constant value, while that of G6/ADP exhibited a steep decrease. Importantly, when the CaCl_2_ concentration was fixed at 1.0 mM, the FL response to ATP is absolutely larger than that to ADP (Fig. 5c and S10 in ESI†). From these results, one can conclude that the largely improved selectivity is ascribed to the chelation of diphosphate to Ca^2+^,^21^ which can strongly inhibit the binding of G6 to ADP and eventually lead to the remarkably high ATP selectivity. The expanded detection range toward ATP is rationalized in terms of the competitive interactions^22^ between guanidinium–phosphate binding and the greater amount of NaCl salt. In other words, to overcome the competitive inhibition, a much higher concentration of ATP is required to bind G6 and to drive self-assembly, consequently exhibiting FL emission. Therefore, we have gained a new perspective that the selectivity and the detection range under the turn-on characteristics are controllable, through the strategy of the binding mechanisms operating in the self-assembly process, which enables us to tailor a new assembly-based FL sensory system.

## Conclusions

In conclusion, we demonstrated that G6 has superior tolerance to self-aggregation and exhibits preferential selectivity to ATP over ADP under salt conditions. Of greatest importance is the finding that by utilizing the salt interference one can adjust the guanidinium–phosphate binding in the self-assembly process and can achieve the tailoring of the high selectivity and the broad detection range as well as the turn-on characteristics. This study revealed that the amplification cascade in the assembly-based sensory system skillfully utilizes the binding–self-assembly–FL response relationship. Among them, the binding mechanism in the self-assembly process plays a pivotal role in determining the performance level of the sensory system. This can be achieved only when the binding event is taken into consideration with the self-assembly process. As seen in biological systems, the binding event is a universal trigger to orchestrate a whole assembly-based system. We believe, therefore, that our study has opened up a new avenue to design assembly-based FL chemosensors capable of sensing an analyte of interest in an appropriate detection range. We envision that the concept of this assembly-based sensory system will realize the FL visualization of dynamic biological events: for example, the production and the consumption of ATP occurring in a live cell.
