# Supplementary material for: Tailoring of the desired selectivity and the turn-on detection range in a self-assembly-based fluorescence sensory system
Source: Chem Sci. 2015 May 6;6(7):3863–7. doi: 10.1039/c5sc00863h (PMC5707478; doi:10.1039/c5sc00863h)
Supplement: Supplementary file 1 [file SC-006-C5SC00863H-s001.pdf]

Electronic Supplementary Material (ESI) for Chemical Science.
This journal is © The Royal Society of Chemistry 2015

Electronic Supplementary Information

Tailoring of the desired selectivity and the turn-on detection range in a self-assembly-based fluorescence sensory system

Takao Noguchi,*[ab] Bappaditya Roy,[a] Daisuke Yoshihara,[b] Youichi Tsuchiya,[b] Tatsuhiro Yamamoto[b] and Seiji Shinkai*[abc]

[a]*Institute for Advanced Study, Kyushu University, 744 Moto-oka, Nishi-ku, Fukuoka 819-0395, Japan.* [b]*Nanotechnology Laboratory, Institute of Systems, Information Technologies and Nanotechnologies (ISIT), 4-1 Kyudai-Shinmachi, Nishi-ku, Fukuoka 819-0388, Japan.* [c]*Department of Nanoscience, Faculty of Engineering, Sojo University, 4-22-1 Ikeda, Kumamoto 860-0082, Japan.*

***Corresponding author**.
E-mail: shinkai_center@mail.cstm.kyushu-u.ac.jp; tnoguchi@mail.cstm.kyushu-u.ac.jp

Scheme S1 Synthetic route to G2, G4 and G6.

Fig. S1 Dependence of volume fraction of DMSO on the solubility of G2 and G6.

Fig. S2 (a) Excitation and fluorescence spectra of G4 in the presence of ATP. (b) UV-Vis spectra of G4 in the absence and the presence of ATP.

Fig. S3 Fluorescence titration of G2 and G6 upon addition of increasing concentrations of AMP, ADP and ATP.

Fig. S4 UV-Vis titration of (a) G2 and (b) G6 with ATP.

Fig. S5 Fluorescence images of the dispersion of (a) G2 and (b) G6 upon addition of ATP.

Fig. S6 Concentration dependence of (a) G4 and (b) G6 on fluorescence emission under the physiological salt condition.

Fig. S7 Fluorescence titration of G6 upon addition of increasing concentrations of AMP, ADP and ATP under the physiological salt condition.

Fig. S8 Time-dependence on the fluorescence response of G6 to ATP under the physiological salt condition.

Fig. S9 ATPase dependence on FL emission from G6/ATP.

Fig. S10 Fluorescence titration of G6 upon addition of increasing concentrations of AMP, ADP and ATP with the fixed concentration of $CaCl_2$ (1.0 mM).

Materials.

All commercially available chemicals are of reagent grade and used as received. Solvents used for synthesis are of super dehydrated grade. $CDCl_3$ and DMSO-d_6 containing 0.03 v/v% TMS for NMR were purchased from ACROS ORGANICS. Adenosine 5'-monophosphate (AMP) disodium salt, adenosine 5'-diphosphate (ADP) disodium salt and adenosine 5'-triphosphate (ATP) disodium salt trihydrates were purchased from Wako Pure Chemical, Ltd. ATPase (Cat. A7510) was purchased from Sigma-Aldrich Chem. Co. Distilled water was purified with a SimPack® 1 (Millipore, Co.).

Measurements.

^{1}H NMR (400 MHz) and ^{13}C NMR (100 MHz) were recorded on a JEOL JNM-ECS400 spectrometer. Chemical shifts were reported in ppm with the signals of TMS as an internal standard for ^{1}H NMR and residual solvent for ^{13}C NMR measurements. Matrix-assisted laser desorption ionization time-of-flight (MALDI-TOF) mass spectrum was obtained by Bruker autoflex speed. Electrospray ionization time-of-flight (ESI-TOF) mass spectrum was obtained by Bruker MicroTOF. UV-Vis absorption was recorded on a JASCO V-670 equipped with a peltier-type thermostatic cell holder. A quartz cell with 1 cm path length was used for UV-Vis absorption measurements. Fluorescence spectra were recorded by Perkin-Elmer LS55 luminescence spectrophotometer using quartz cells with 1 mm path lengths. Fluorescence microscopic observation was performed by using Leica DM2500 (λ_{ex} = 340–380 nm).

Fluorescence measurements.

The titration experiments with the nucleotides (AMP, ADP and ATP) were performed with a solution (1.5 mL) of 6.0 μM OPV and nucleotides (varied concentrations) in 10 mM HEPES (pH 7.4). The physiological salt condition is as follows: 125 mM NaCl, 5.0 mM KCl, 1.0 mM $CaCl_2$, 0.5 mM $MgCl_2$.

ATPase dependence on FL emission from G6/ATP was investigated by using TECAN infinite M200PRO. In a 96-well flat bottom plate, 1.25 units/mL, 0.625 units/mL and 0.313 units/mL of ATPase solutions (10 μL for the three) were added separately to 30 μM ATP solutions (90 μL). After incubation at 37 ^{o}C for 20 min, 110 μM G6 solution (10 μL) was added and then fluorescence measurement was performed. For the sample preparation, the following buffer solution was used: 10 mM NaCl, 5.0 mM KCl, 0.5 mM $CaCl_2$, 0.5 mM $MgCl_2$, 10 mM HEPES (pH 7.4).

Scheme S1. Synthetic route to G2, G4 and G6.

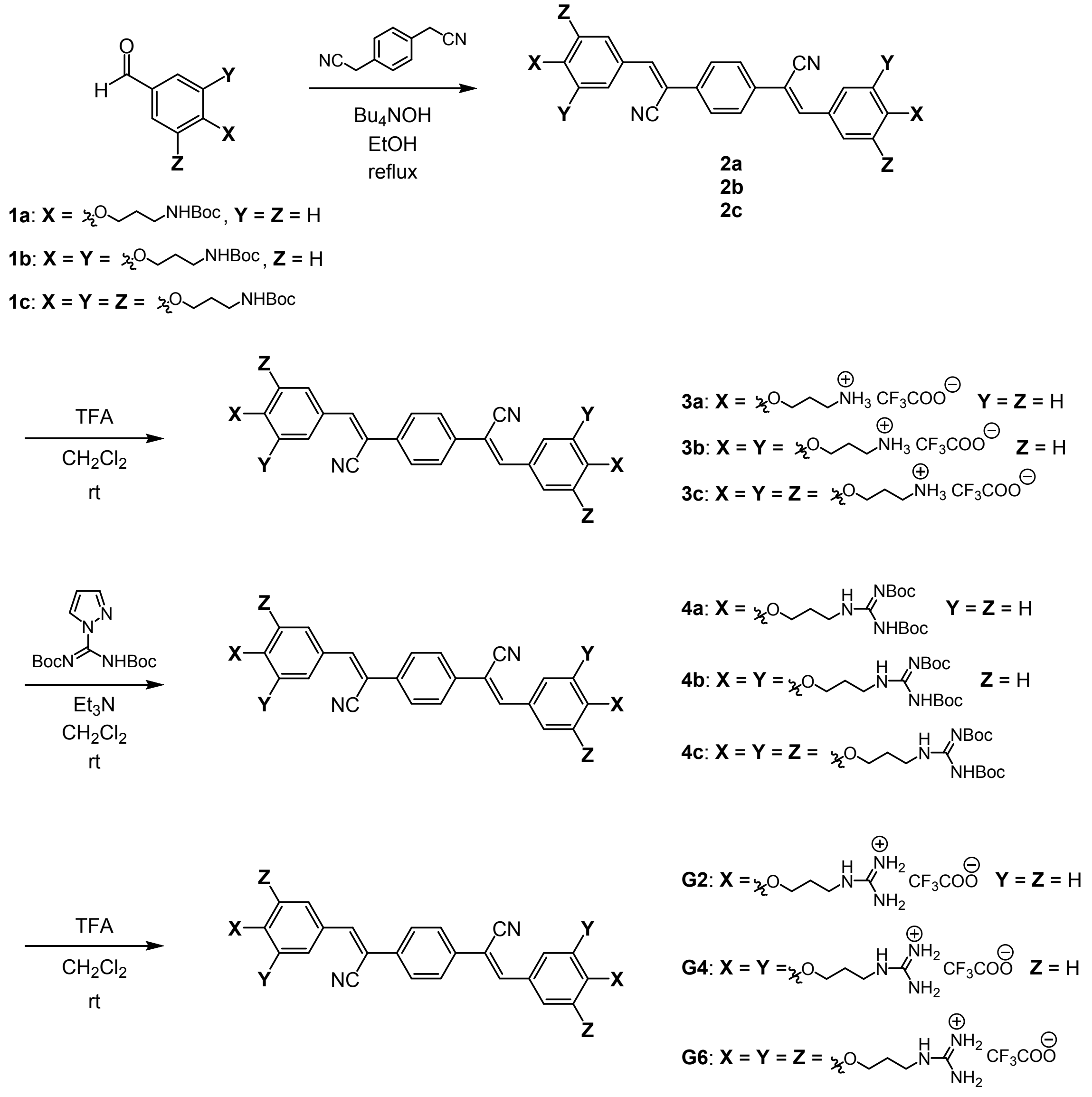

G2, G4 and G6 were synthesized according to the previously reported procedure (ref. 14).

G2: Hygroscopic yellow solid.
^{1}H NMR (400 MHz, DMSO-d_6): δ 8.08 (s, 2H), 7.99 (d, J = 9.2 Hz, 4H), 7.86 (s, 4H), 7.63 (t, J = 5.4 Hz, 2H), 7.14 (d, J = 9.2 Hz, 4H), 7.13 (br-s, 8H), 4.13 (t, J = 6.2 Hz, 4H), 3.30 (q, J = 6.2 Hz, 4H), 1.97 (quint, J = 6.2 Hz, 4H).
^{13}C NMR (100 MHz, DMSO-d_6): δ 160.5, 159.0 (q, ^{2}J = 32 Hz, CF_3***C***OO), 142.8, 134.3, 131.3, 126.3, 126.1, 118.2, 117.0 (q, ^{1}J = 297 Hz, ***C***F_3COO), 115.0, 106.3, 65.1, 37.8, 28.1.
MS (MALDI): m/z 563.1 ([*M*-H^+−2CF_3COO^-]$^+$).
Elemental Analysis: calcd. (%) for $C_{36}H_{36}F_6N_8O_6$ (G2): C 54.68, H 4.59, N 14.17; found: C 54.30, H 4.55, N 14.07.

G4: Highly hygroscopic yellow solid.
^{1}H NMR (400 MHz, DMSO-d_6): δ 8.07 (s, 2H), 7.86 (s, 4H), 7.78 (t, J = 5.4 Hz, 2H), 7.77 (t, J = 5.4 Hz, 2H), 7.73 (d, J = 2.0 Hz, 2H), 7.61 (dd, J = 8.8, 2.0 Hz, 2H), 7.21 (br-s, 16H), 7.20 (d, J = 8.8 Hz, 2H), 4.13 (t, J = 6.4 Hz, 4H), 4.10 (t, J = 6.4 Hz, 4H), 3.28 (m, 8H), 2.00 (quint, J = 6.4 Hz, 4H), 1.98 (quint, J = 6.4 Hz, 4H).
^{13}C NMR (100 MHz, DMSO-d_6): δ 159.2 (q, ^{2}J = 32 Hz, CF_3***C***OO), 157.1, 150.6, 147.9, 143.1, 134.4, 126.6, 126.1, 124.1, 118.3, 117.0 (q, ^{1}J = 297 Hz, ***C***F_3COO), 114.1, 113.4, 106.4, 65.7, 65.6, 37.74, 37.66, 28.2, 28.1.
MS (MALDI): m/z 793.0 ([*M*-3H^+−4CF_3COO^-]$^+$).
HRMS (ESI): m/z calcd. for [*M*−CF_3COO^-]$^+$: 1135.4155; found: 1135.4157.

G6: Highly hygroscopic yellow solid.
^{1}H NMR (400 MHz, DMSO-d_6): δ 8.09 (s, 2H), 7.88 (s, 4H), 7.86 (t, J = 5.6 Hz, 4H), 7.75 (t, J = 5.6 Hz, 2H), 7.40 (s, 4H), 7.25 (br-s, 24H), 4.09 (t, J = 6.4 Hz, 8H), 4.05 (t, J = 6.4 Hz, 4H), 3.35 (q, J = 6.4 Hz, 4H), 3.30 (q, J = 6.4 Hz, 8H), 2.01 (quint, J = 6.4 Hz, 8H), 1.88 (quint, J = 6.4 Hz, 4H).
^{13}C NMR (100 MHz, DMSO-d_6): δ 159.2 (q, ^{2}J = 32 Hz, CF_3***C***OO), 157.0, 152.2, 143.3, 139.2, 134.4, 129.0, 126.3, 118.0, 116.9 (q, ^{1}J = 297 Hz, ***C***F_3COO), 108.2, 108.1, 70.2, 65.8, 37.9, 37.7, 29.3, 28.2.
MS (MALDI): m/z 1023.2 [*M*-5H^+−6CF_3COO^-]$^+$.
HRMS (ESI): m/z calcd. for [*M*−CF_3COO^-]$^+$: 1593.5503; found: 1593.5510.

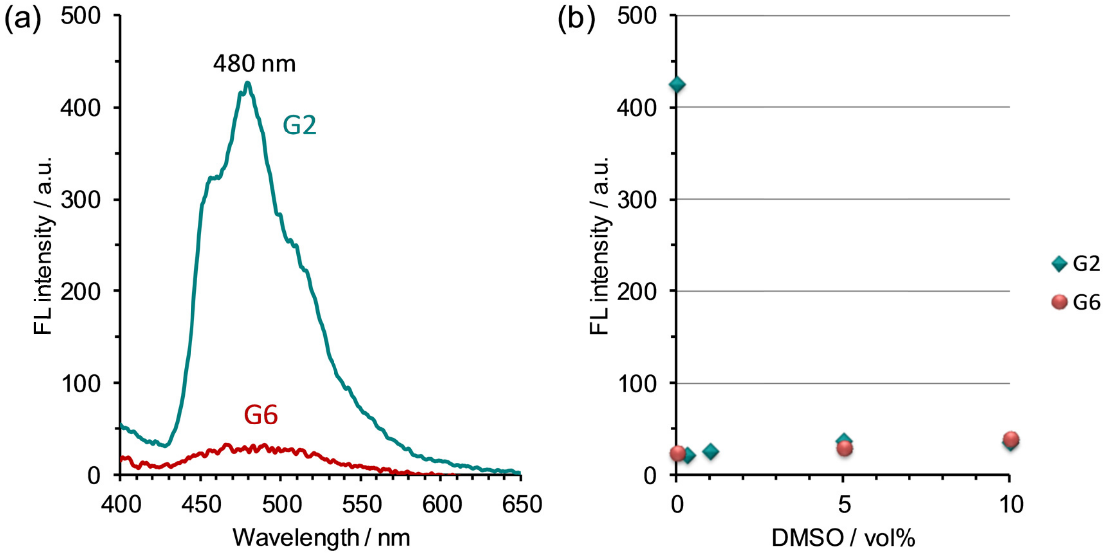

Fig. S1 Dependence of volume fraction of DMSO on the solubility of G2 (green) and G6 (red). (a) Fluorescence spectra of G2 and G6 in water. (b) Changes in the FL intensity of G2 and G6 upon increasing volume fractions of DMSO in water.

Conditions: [G2] = [G6] = 6.0 μM; [HEPES] = 10 mM (pH 7.4); 25 °C; λ_{ex} = 388 nm.

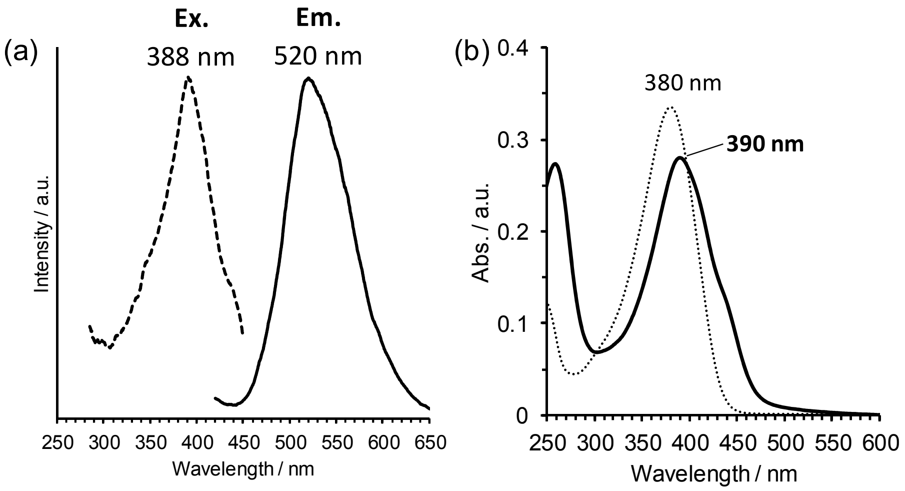

Fig. S2 (a) Excitation (λ_{em} = 520 nm) and fluorescence spectra (λ_{ex} = 388 nm) of G4 in the presence of ATP (20 μM) in DMSO/water (1:9 v/v). (b) UV-Vis spectra of G4 in the absence (dotted line) and the presence of ATP (solid line) in DMSO/water (1:9 v/v).

Conditions: [G4] = 6.0 μM; [ATP] = 20 μM; [HEPES] = 10 mM (pH 7.4); 25 °C.

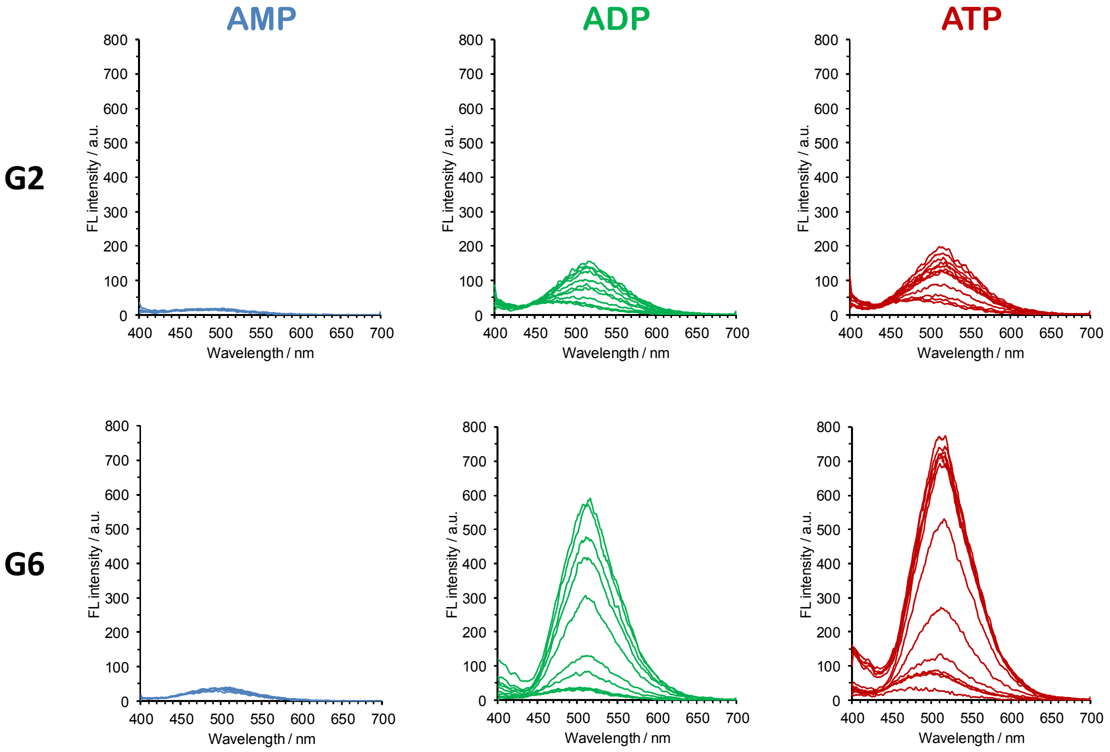

Fig. S3 Fluorescence titration of G2 (upper) and G6 (bottom) upon increasing concentrations of AMP (blue), ADP (green) and ATP (red) in DMSO/water (1:9 v/v).

Conditions: [G2] = [G6] = 6.0 μM; [HEPES] = 10 mM (pH 7.4); 25 °C; λ_{ex} = 388 nm; λ_{em} = 514 nm.

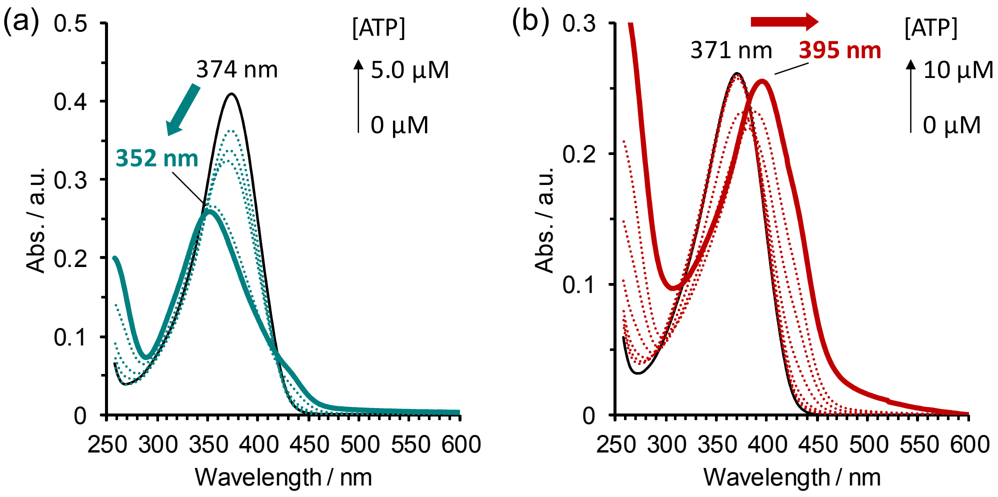

Fig. S4 UV-Vis titration of (a) G2 and (**b**) G6 with ATP in DMSO/water (1:9 v/v).
Conditions: [G2] = [G6] = 6.0 μM; [HEPES] = 10 mM (pH 7.4); 25 °C.

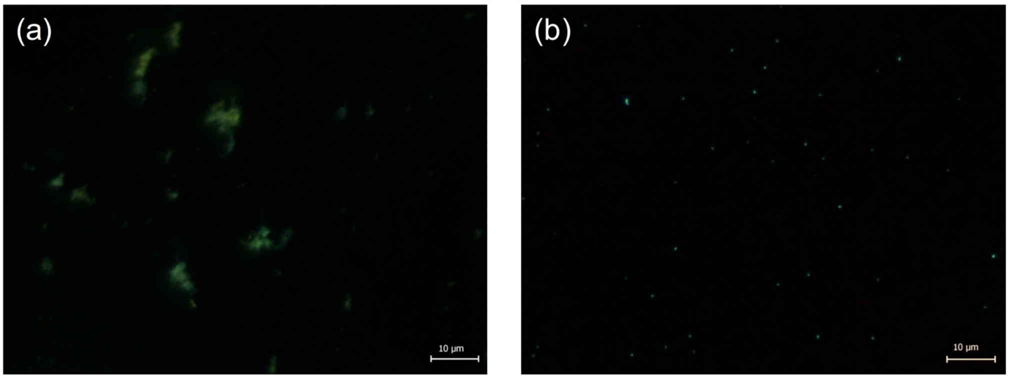

Fig. S5 Fluorescence images of the dispersion of (a) G2 and (b) G6 upon addition of ATP (20 μM) in DMSO/water (1:9 v/v).
Conditions: [G2] = [G6] = 6.0 μM; [HEPES] = 10 mM (pH 7.4); 25 °C; λ_{ex} = 340–380 nm.

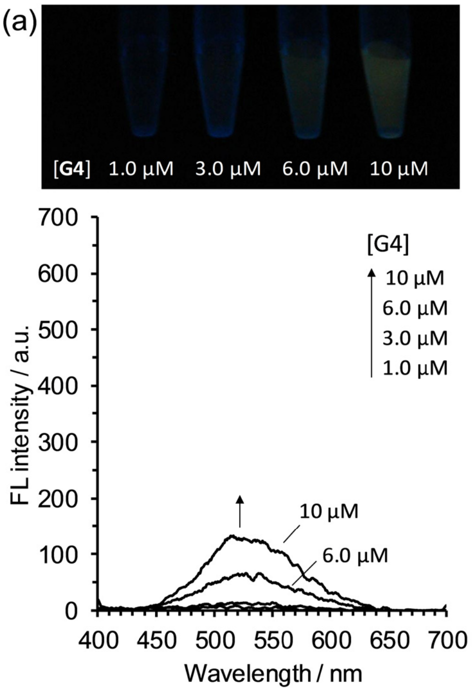

Fig. S6 Concentration dependence of (a) G4 and (b) G6 on fluorescence emission under the physiological salt condition. Photographs are the corresponding fluorescence images upon irradiation of UV light (λ_{ex} = 365 nm).

Conditions: [NaCl] = 125 mM; [KCl] = 5.0 mM; [$CaCl_2$] = 1.0 mM; [$MgCl_2$] = 0.5 mM; [HEPES] = 10 mM (pH 7.4); 25 °C; λ_{ex} = 388 nm.

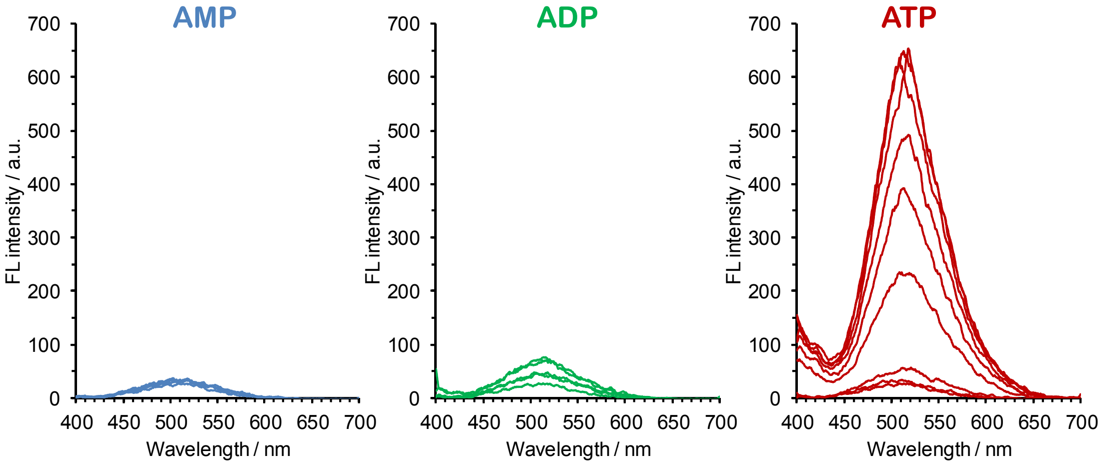

Fig. S7 Fluorescence titration of G6 upon addition of increasing concentrations of AMP (blue), ADP (green) and ATP (red) under the physiological salt condition.

Conditions: [G6] = 6.0 µM; [NaCl] = 125 mM; [KCl] = 5.0 mM; [$CaCl_2$] = 1.0 mM; [$MgCl_2$] = 0.5 mM; [HEPES] = 10 mM (pH 7.4); 25 °C; λ_{ex} = 388 nm; λ_{em} = 514 nm.

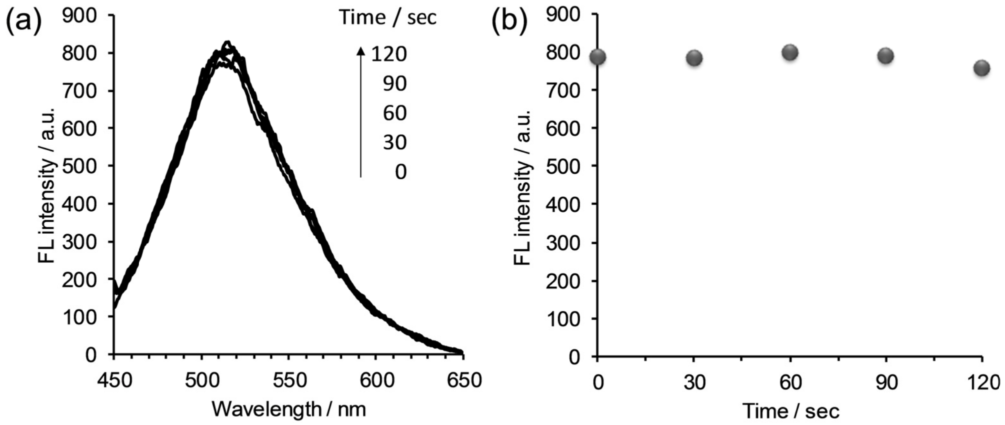

Fig. S8 Time-dependence on the fluorescence response of G6 (6.0 μM) to ATP (180 μM) under the physiological salt condition. (a) Time course of the fluorescence spectrum and (b) the fluorescence intensity.

Conditions: [NaCl] = 125 mM; [KCl] = 5.0 mM; [$CaCl_2$] = 1.0 mM; [$MgCl_2$] = 0.5 mM; [HEPES] = 10 mM (pH 7.4); 25 °C; λ_{ex} = 388 nm; λ_{em} = 514 nm.

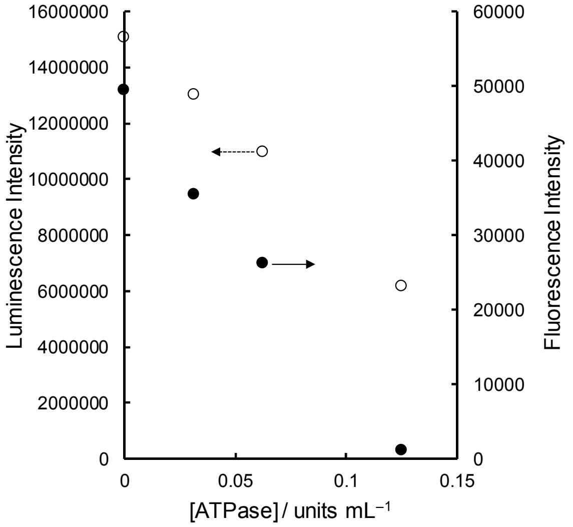

Fig. S9 ATPase dependence on FL emission from G6/ATP (filled circle: G6/ATP, right axis; open circle: commercially available ATP indicator, cellTiter-Glo®, left axis).

Conditions: [G6] = 10 μM; [ATP] = 24.5 μM; [NaCl] = 10 mM; [KCl] = 5.0 mM; [$CaCl_2$] = 0.5 mM; [$MgCl_2$] = 0.5 mM; [HEPES] = 10 mM (pH 7.4); λ_{ex} = 380 nm; λ_{em} = 510 nm.

Although ATP still exists as indicated by a commercially-available ATP indicator (open circles in Fig. S9), the FL intensity of G6 disappears at [ATPase] = 0.125 units mL^{-1} (filled circles in Fig. S9) because of the threshold characteristic of the self-assembly system (refs. 12 and 14).

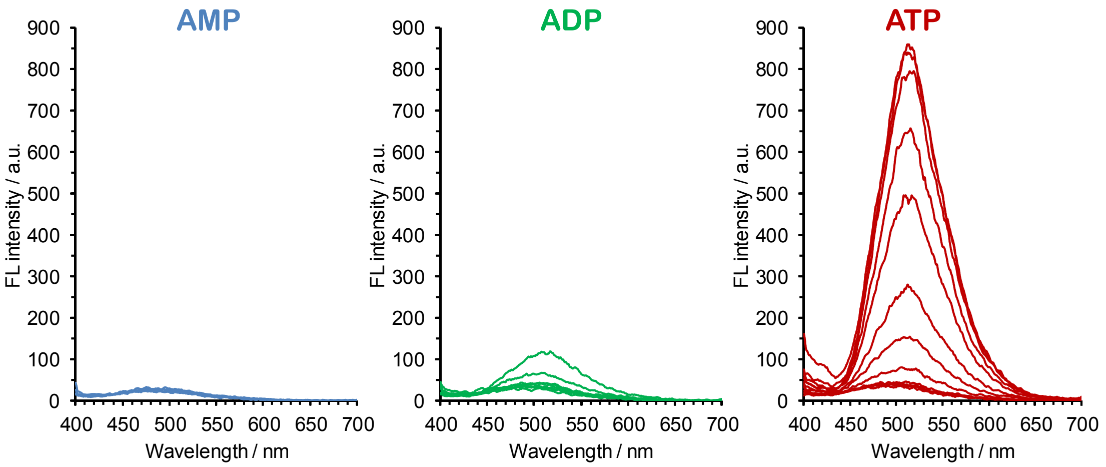

Fig. S10 Fluorescence titration of G6 upon addition of increasing concentrations of AMP (blue), ADP (green) and ATP (red) with the fixed concentration of $CaCl_2$ (1.0 mM).
Conditions: [G6] = 6.0 μM; [HEPES] = 10 mM (pH 7.4); 25 °C; λ_{ex} = 388 nm; λ_{em} = 514 nm.
